# Optimized radiotherapy to improve clinical outcomes for locally advanced lung cancer

**DOI:** 10.1186/s13014-018-1094-y

**Published:** 2018-08-13

**Authors:** Nicolas Jaksic, Enrique Chajon, Julien Bellec, Romain Corre, Charles Ricordel, Bertrand de Latour, Hervé Lena, Ulrike Schick, Renaud de Crevoisier, Joël Castelli

**Affiliations:** 1 Département de Radiothérapie, Centre Eugène Marquis, Rue de la Bataille Flandres Dunkerque, Rennes, France; 2 Université de Rennes 1, Rennes, France; 3 Service de pneumologie CHU Pontchaillou, Rennes, France; 4 Service de chirurgie thoracique, Clinique Saint Laurent, Rennes, France; 5 Département de Radiothérapie, CHRU Brest, Brest, France

**Keywords:** NSCLC, IMRT, SMART, Radiotherapy, Acceleration

## Abstract

**Background:**

We aimed to evaluate the toxicity, loco-regional control (LRC) and overall survival (OS) associated with accelerated intensity-modulated radiotherapy (IMRT) for locally advanced lung cancer.

**Methods:**

Seventy-three patients were consecutively treated with IMRT from November 2011 to August 2016. A total dose of 66 Gy was delivered using two different schedules of radiotherapy: simultaneous modulated accelerated radiotherapy (SMART) (30 × 2.2 Gy, across 6 weeks) with or without chemotherapy, or moderate hypofractionated radiotherapy (HRT) (24 × 2.75 Gy, across 4 weeks) in patients unfit to receive concomitant chemotherapy. Data on esophageal and pulmonary toxicities, LRC and OS were prospectively collected.

**Results:**

The median follow-up duration was 44 months. Severe pneumonitis and esophagitis (grade 3–4) were observed in 7% and 1% of patients respectively, with only one case of grade 4 (pneumonitis). Overall, the 1-year and 2-year LRCs were 76% [95 confidence interval (CI)%: 66–87%] and 62% [95 CI%: 49–77%] respectively. The 1 and 2-year OS rates were 72% [95% CI: 63–83%] and 54% [95 CI%: 43–68%] respectively. None parameters were correlated with LRC or OS. In particular, no difference was observed between patients treated with SMART and H-RT (*p* = 0.26 and 0.6 respectively), with a 1-year LRC of 74% [95 CI%: 62–86%] for SMART and 91% [95 CI%: 74–100%] for H-RT. No significant differences were observed in the toxicity rates associated with each of the RT schedules.

**Conclusions:**

Accelerated IMRT for locally advanced lung cancer is associated with low toxicities and high LRC. Moderate hypofractionated RT, by decreasing the total treatment time, may be promising in improving clinical outcomes.

## Background

Radiotherapy (RT), at a total dose of 60–66 Gy over 6 weeks, combined with platinum-based chemotherapy [1, 2], is the standard of care for stage IIIB or IIIA patients with unresectable or inoperable disease [3]. However, the long-term outcomes are poor, with a 5-year overall survival (OS) rate of 15–35% for stage IIIA and 5–10% for stage IIIB patients [4].

RT dose escalation has been tested in several phase I and II trials with promising results in terms of tumour control and toxicity [5]. However, the RTOG 0617 phase III randomized trial showed a better median OS (28.7 months) in the standard dose arm (60 Gy in 30 fractions) than in the high dose arm (74 Gy in 37 fractions, 20.3 months) [6]. This unintended and negative result may be attributed to the greater cardiopulmonary toxicity associated with dose escalation and prolonged overall treatment time (OTT). Indeed, a loss of 1.6% per day in the absolute 3-year survival rate is observed, if the OTT extends beyond 6 weeks, due to rapid tumour repopulation [7]. Thus, to improve tumour control, different modifications of fractionation have been tested, with a meta-analysis showing a modest but significant benefit for RT schedules with reduced OTT [8]. However, acceleration using ancient RT techniques is associated with prohibitive toxicities, especially in the case of concomitant chemotherapy, limiting the deployment of these protocols [9].

Compared to 2D and 3D-RT, intensity-modulated radiotherapy (IMRT) allows for improved organs-at-risk sparing, owing to the high dose conformation to the target volume, thus reducing toxicity rates [10].

In this context, the aim of this study was to evaluate the clinical outcomes (toxicity, loco-regional control (LRC) and OS) in patients with locally advanced non-small cell lung cancer (LA-NSCLC) treated with an accelerated RT approach using IMRT.

## Methods

### Patients’ characteristics

From November 2011 to August 2016, 73 consecutive patients with unresectable NSCLC (7th edition AJCC staging [11]) treated with IMRT were retrospectively reviewed.

The main patient characteristics are shown in Table 1. Fifty-seven patients had stage III, and eight patients stage IV tumours. Eight supplementary patients with tumours of stages I-II were included in the analysis. These patients were deemed unfit for surgery and stereotactic body radiation therapy (SBRT) due to the tumour size or localization.

**Table 1:** Demographic and clinicopathological characteristics of the patients

*Characteristic*	*SMART (n = 59)*		*H-RT (n = 14)*		*Total (n = 73)*	
Age (years)						
Mean range	65		65		66	
Sex						
Male	44	75%	9	64%	53	73%
Mutation						
EGFR	4	7%	1	7%	5	7%
Smoking antecedent						
Yes	40	68%	10	71%	50	68%
Histologic type						
Adenocarcinoma	31	53%	8	57%	39	53%
Squamous	24	41%	6	43%	30	41%
Other	4	7%	0	0%	4	5%
AJCC stage						
Stage II	5	9%	3	21%	8	11%
Stage IIIA	40	68%	3	21%	43	59%
Stage IIIB	12	20%	2	14%	14	19%
Stage IV	2	3%	6	43%	8	11%
Chemotherapy type						
No chemotherapy	4	7%	6	43%	10	14%
Sequential chemotherapy	11	19%	8	57%	19	26%
Concurrent chemotherapy	44	75%	0	0%	44	60%
Chemotherapy agent						
Cisplatin-Vinorelbine	27	46%	0	0%	27	37%
Carboplatin-Taxol	19	32%	1	7%	20	27%
Other	7	12%	9	64%	16	22%
PTV volume (cm^3^)						
PTV 66 Gy	287		337		335	
PTV 54 Gy	598				598	

*SMART* simultaneous modulated accelerated radiotherapy, *H-RT* moderate hypofractionated radiotherapy, *EGFR* epidermal growth factor receptor, *AJCC* American Joint Committee on Cancer, *PTV* planning target volume

All patients underwent a physical examination, chest, abdomen and pelvis computed tomography (CT) scan, fluorine-18 fluorodeoxyglucose positron emission tomography CT (18F-FDG-PET/CT), bronchoscopy, and pulmonary function tests at the time of diagnosis.

Diagnosis was histologically confirmed using bronchoscopic or percutaneous fine needle aspiration biopsy. When necessary for treatment decision, a lymph node biopsy guided by an endoscopic ultrasound was indicated in order to confirm the N2–N3 status. The study was approved by the institutional ethical committee.

### Planning computed tomography (CT) and target volume delineation

All patients were immobilized in a supine position. For each patient, a respiratory-correlated planning 4D CT with contrast injection was performed in five respiratory phases. The gross tumour volume (GTV), defined as the primary tumor and clinically positive lymph nodes either on the planning CT (> 1-cm short axis diameter) or pre-treatment 18F-FDG-PET/CT (standardized uptake value > 3), was delineated on each respiratory phase. The internal target volume (ITV) was obtained for the primary tumour from the five phases of the 4D-CT. The clinical target volume (CTV) was defined for the primary tumour as the ITV plus a 5-mm 3D margin to account for microscopic extension (CTV_1). The lymph node levels containing positive lymph nodes (selective nodal irradiation), and lymph node levels adjacent to the invaded nodal stations were considered the CTV for the second dose level (CTV_2). Elective treatment of the mediastinum and supraclavicular fossae was not allowed. A 7-mm 3D margin was used to create the planning target volume (PTV_1 and PTV_2).

### Radiotherapy schedules and planning

Two different IMRT schedules were used, simultaneous modulated accelerated radiotherapy (SMART), or moderate hypofractionated radiotherapy (H-RT). In the SMART schedule, two dose levels were used. A total dose of 66 Gy (2.2 Gy per day) was prescribed for PTV_1 (high-risk level) and 54 Gy (1.8 Gy per day) for PTV_2 (low-risk level). The prescribed doses were delivered simultaneously in 30 fractions, 5 days a week, over 6 weeks, with or without concomitant platinum-based chemotherapy. Patients received mainly three to four cycles of cisplatin (80 mg/m^2^ day 1) and vinorelbine (25 mg/m^2^ day 1 and 8) every three weeks or six cyles of carboplatin (AUC 2) and paclitaxel (45 mg/m^2^) weekly. Radiotherapy started concurrently with the second or third cycle.

In the H-RT schedule, only one dose level was used. The same steps as previously described were followed to define the CTV, ITV and PTV. A total dose of 66 Gy (2.75 Gy per day) was prescribed for PTV_1 alone (high-risk level). No low-risk level was used. The prescribed dose was delivered in 24 fractions, 5 days a week, over 4 weeks. This schedule was introduced in February 2014, in patients unfit for chemotherapy (concomitant or sequential).

Data on the dose constraints associated with the two schedules are shown Table 2. For each patient, Pinnacle v9 treatment planning system (Philips Medical Systems, Best, Netherlands) was used to create a treatment plan comprising five modulated 6 MV photon beams from an Elekta Synergy linear accelerator (Elekta, Crawley, the UK). Treatment delivery was performed using a step-and-shoot IMRT technique. Direct Machine Planning Optimization (DMPO) inverse planning algorithm (Philips Medical Systems, Best, Netherlands) was used to optimize the beams’ fluence. A collapsed cone convolution superposition dose calculation algorithm was used for suitable heterogeneity correction.

**Table 2:** Dose constraints to organs at risk (OAR)

Organ at risk	Dose constraints
Total normal lung volume excluding the CTV	D mean < 20 Gy
	V20 < 30%
	V30 < 20%
	V5 < 65%
Heart	V30 < 50%
PRV Spinal cord	D2% < 45 Gy
PRV Oesophagus	D2% < 66 Gy
	V60 < 10%
	V50 < 30%

*CTV* clinical target volume, *D mean* mean dose, *PRV* planning organ at risk volume, *Vx* percentage of the total organ volume receiving ≥ xGy

### Image-guided radiotherapy protocol

An off-line image-guided RT (IGRT) protocol was used. Cone beam CT images were obtained daily on the first 3 days and then weekly [12]. Region-of-interest for matching was set to encompass the carina, adjacent vertebral bodies and the GTV. After registration, the translational corrections were applied to the treatment couch. If all the variances were < 5 mm, the treatment proceeded without correction. If one or more corrections were > 5 mm, adjustment was necessary prior to treatment, and daily imaging with online corrections was allowed.

### Data collection and statistics

During treatment, a weekly physical examination was performed. Chest radiography was performed 6 weeks after treatment completion. Tumour response was evaluated using a 18F-FDG-PET/CT scan at 3 months after the end of RT. In case of partial response, a second 18-FDG-PET/CT was performed 3 months later. In the case of complete response, the follow-up comprised a CT scan of the chest and abdomen every 3 months. Toxicities were assessed in terms of duration and severity. Acute oesophageal toxicity was recorded as the maximum severity of acute oesophagitis and acute lung toxicity as the first recorded treatment-related pneumonitis experienced. Toxicity was scored according to the Common Terminology Criteria for Adverse Events (CTCAE) 4.0.

Loco-regional recurrence was defined as the progression of the target lesion or the appearance of a new lesion in the previous irradiated volume. LRC was calculated from the first day of RT to the date of the first loco-regional recurrence. Overall survival (OS) was calculated from the first day of RT to the date of death from any cause. Patients alive at the time of analysis were censored at the date of the last follow-up. Follow-up was calculated using reverse Kaplan-Meier estimation [13]. LRC and OS estimations were computed using the Kaplan-Meier method, and a two-sided log-rank test was used to compare groups. The association of the pre-treatment parameters with LRC and OS was assessed using Cox analyses. A Chi-square test was used to compare the toxicity rates of the schedules. All analyses were performed using R software 3.2.4 (R Development CoreTeam; http://www.r-project.org). The database was locked on October 1, 2017.

## Results

The median follow-up was 44 months. At the time of analysis, 43 patients were dead, 42 had systemic recurrences, and 22 had loco-regional recurrences. Fifty-nine patients were treated using the SMART schedule, and fourteen using the H-RT schedule. Tumour coverage and dose constraints to the OARs (organs at risk) were respected, and there was no relevant difference between the two schedules (Table 3).

**Table 3:** Mean dosimetric data by radiotherapy schedule

Organ at risk		SMART (SD)	H-RT (SD)	*p*-value
Lungs-PTV	Mean dose (Gy)	13.9 (11.4–16.3)	11.5 (8.8–14.1)	< 0.01
	V5 (%)	59.6 (48.0–71.2)	50.8 (40.8–60.7)	< 0.01
	V20 (%)	25.9 (20.2–31.6)	20.2 (14.3–26.2)	< 0.01
	V30 (%)	16.3 (11.9–20.8)	13.1 (8.6–17.5)	0.01
Ipsilateral lung	Mean dose (Gy)	24.5 (18.3–30.8)	22.7 (16.0–29.3)	NS
	V5 (%)	69.3 (53.6–85.1)	61.9 (48.5–75.3)	NS
	V20 (%)	60.0 (38.0–63.9)	45.9 (33.8–58.0)	NS
	V30 (%)	37.1 (24.0–50.1)	33.8 (20.3–47.2)	NS
Contralateral lung	Mean dose (Gy)	9.1 (5.2–13.1)	10.8 (0–26.3)	0.02
	V5 (%)	55.4 (39.3–71.5)	48.4 (40.0–56.7)	NS
	V20 (%)	11.9 (3.0–20.8)	3.3 (0.1–6.4)	< 0.01
	V30 (%)	7.2 (0–15.8)	0.9 (0–2.4)	< 0.01
Heart	V30 (%)	12.8 (0.0–27.2)	11.1 (0.0–25.8)	NS
	V40 (%)	6.6 (0.0–14.2)	4.8 (0.0–11.7)	NS
	Mean dose (Gy)	10.5 (1.8–19.2)	8.9 (0.3–17.5)	NS
Oesophagus	V50 (%)	33.2 (18.7–47.7)	17.7 (8.7–26.7)	< 0.01
	V60 (%)	11.1 (0.0–22.4)	8.4 (1.8–15.0)	NS

*SD* Standard deviation, *Vx* percentage of the total organ volume receiving ≥ xGy, *SMART* simultaneous modulated accelerated radiotherapy, *H-RT* moderate hypofractionated radiotherapy, *NS* non significant

### Toxicity

Data on the toxicities are described in Table 4. The most prevalent type of toxicity was oesophagitis. Twenty-five patients (34%) had grade 2, and 1 patient (1%) had grade 3 oesophagitis; the latter required hospitalization and nasogastric tube feeding for nutritional support. Ten patients (13%) had grade ≥ 2 pneumonitis, and one (1%) had grade 4 pneumonitis. There was no significant difference between the different irradiation schedules (*p* = 0.2).

**Table 4:** Radiotherapy related acute adverse events

	SMART (*n* = 59)	H-RT (*n* = 14)	*P* value	Total (*n* = 73)
Pneumonitis				
Grade 2	5 (8%)	0 (0%)	NS	5 (7%)
Grade 3	3 (5%)	1 (7%)	NS	4 (5%)
Grade 4	1 (2%)	0 (0%)	NS	1 (1%)
Oesophagitis				
Grade 1	29 (49%)	10 (71%)	NS	39 (53%)
Grade 2	23 (39%)	2 (14%)	NS	25 (34%)
Grade 3	1 (2%)	0 (0%)	NS	1 (1%)

*SMART* simultaneous modulated accelerated radiotherapy, *H-RT* moderate hypofractionated radiotherapy, *NS* non significant

Regarding dosimetrics parameters, the V20 (percentage of the total lungs volume receiving ≥20 Gy) and the V30 for the contralateral lung were significantly higher in the SMART schedule (Table 3). However, the dosimetric constraints were respected for these patients. Moreover, the doses delivered to the lung were not significantly differents for the patients with grade ≥ 2 pneumonitis and with grade ≤ 1 pneumonitis.

A treatment interruption of a mean duration of 5.2 days (ranging from 1 to 8 days) was necessary for six patients treated with concomitant chemotherapy (all of them treated with SMART), mainly due to the presence of oesophagitis (in 3 patients), and to a lesser extent due to other events, such as spontaneous peritonitis, *Clostridium difficile*-associated diarrhoea and epistaxis.

### Loco-regional control and overall survival

**Fig. 1:**
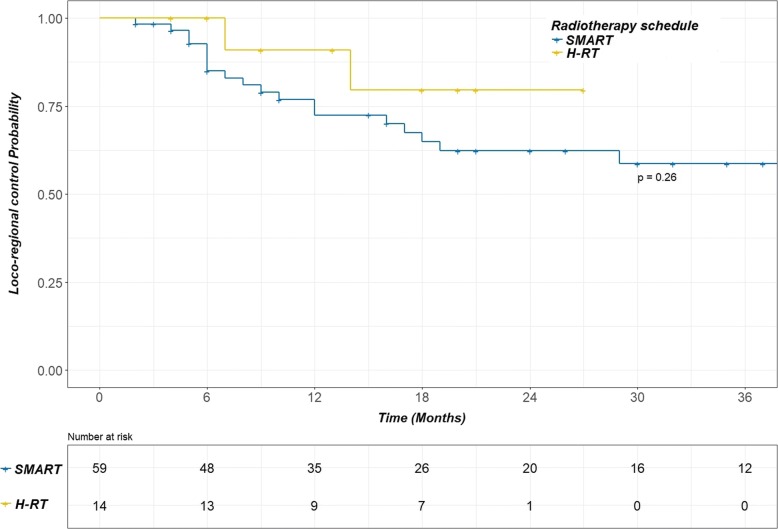
Kaplan Meier curve for loco-regional control comparing the SMART and H-RT schedules. No difference in loco-regional control was observed between the SMART (blue line) and H-RT (yellow line) schedules. SMART: simultaneous modulated accelerated radiotherapy H-RT: moderate hypofractionated radiotherapy

**Fig. 2:**
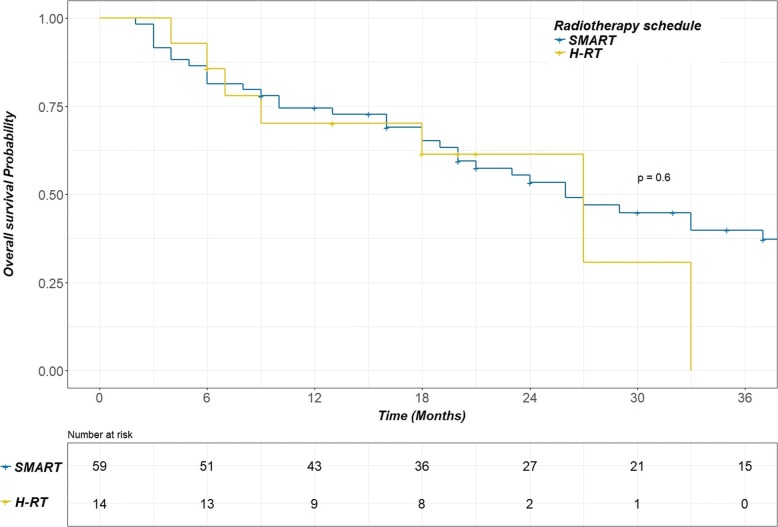
Kaplan Meier curve for overall survival comparing the SMART and H-RT schedules. No difference in overall survival was observed between the SMART (blue line) and H-RT (yellow line) schedules. SMART: simultaneous modulated accelerated radiotherapy. H-RT: moderate hypofractionated radiotherapy

For the whole population, the 1-year, 2-year and 4-year LRCs were 76% [95% confidence interval (95% CI): 66–87%], 62% [95% CI: 49–77%] and 55% [95% CI: 41–75%] respectively. The median time for LRC was not reached. The 1-year, 2-year and 4-year OS rates were 72% [95% CI: 63–83%], 54% [95% CI: 43–68%] and 29% [95% CI: 19–45%] respectively, with a median OS of 27 months. Grade ≥ 2 pneumonitis was the solely parameter correlated with the OS (Hazard ratio = 2.94, *p* = 0.2). The 2-year OS was 15% [95% CI: 3–80%] for patients with Grade ≥ 2 pneumonitis compared to 61% [95% CI: 49–75%] for patients with Grade ≤ 1 pneumonitis. However, no significant difference was shown for LRC. Moreover, oesophageal toxicity and irradiation schedule were not correlated with clinical outcome (Figs. 1 and 2).

## Discussion

In the present study, we described our experience using two accelerated schedules of IMRT in locally advanced lung cancer patients and demonstrated the presence of a low rate of severe oesophageal and pulmonary toxicity, along with encouraging LRC and OS. We hypothesize that our results are associated with the dosimetric advantages of IMRT, and the reduced OTT obtained with the use of accelerated RT schedules.

Accelerated RT, by decreasing the OTT allows for increases in the biologically effective dose (BED). This approach shows an overall survival benefit but there are concerns pertaining to the increased toxicity compared to non-accelerated RT [8, 14]. In our study, thanks to the use of IMRT, the whole dose constraints were respected, in particular for the lung and oesophagus (Table 3). This explains the low rate of grade 3 and 4 pneumonitis (5% and 1% respectively), grade 3 oesophagitis (1%) and the absence of grade 4 oesophagitis. The pulmonary toxicity was correlated with a worse OS in the RTOG 0617 trial [6] and in our study. Interestingly, grade ≥ 2 pneumonitis occurred despite the respect of the dose constraints, highlighting the need of new volume constraint, as the integration of lung perfusion scintigraphy [15].

Compared to 3D-RT, IMRT allows for better sparing of the surrounding normal tissues [16, 17]. Moreover, the use of a 4D-CT for each patient to incorporate the respiratory motion allows to minimize the PTV, leading to a reduction in the dose received by organs at risk [18]. This dosimetric benefit is likely to translate to a decrease in toxicity [19, 20]. Indeed, compared to 3D-RT, IMRT allows to lower rates of severe pneumonitis [21] and a better quality of life [22]. Moreover, the use of IMRT translates to a decrease in the radiation treatment interruption and correlates to a better OS [23].

Another advantage of IMRT relies on the possibility of accelerating the irradiation dose to the high tumour burden alone, while maintaining moderate doses to the anatomical areas considered being at risk of microscopic invasion and areas of overlap between the target and OAR. Retrospective studies in patients with LA-NSCLC reported their experiences with the use of SMART schedules [24–27]. The prevalence of grade 3 oesophagitis and pneumonitis ranged from 0 to 25.3%, and 1% to 11%, respectively, which is in line with our results. The 2-year LRC and OS ranged from 62 to 66%, and from 45 to 56%, respectively. In our cohort, the median OS was 27 months, with a 2-year LRC and OS of 62% and 54%, respectively.

In the present study, a small group was not eligible for concomitant chemotherapy, and these patients were treated with a more accelerated schedule (24 × 2.75 Gy) based on the promising results of a phase II trial evaluating the effect of cetuximab with concurrent chemoradiotherapy for LA-NSCLC. In this trial, patients were treated with a hypofractionated and accelerated schedule (24 × 2.75 Gy), and a concurrent daily low dose of cisplatin (6 mg/m^2^). Despite the non-benefit of cetuximab, the OS was remarkably high, with a median OS of 31.5 months and 1- and 2- year OS rates of 74.5% and 59.4%, respectively. However this benefit is penalized by a high rate of acute and late oesophageal toxicity [28]. For the frail patients in our study we adopted the H-RT schedule to compensate for the inability to receive concomitant chemotherapy. Moreover, this group of patients comprised 43% stage IV (Table 1), with a single metastasis, and having received first-line chemotherapy alone and/or a local treatment of the single metastasis, followed by exclusive chest irradiation only. Despite the poor prognosis of these patients (elderly patients and/or with comorbidities, stage IV) unfit for chemotherapy, the use of H-RT led to an OS and LRC that were similar to those observed in the SMART and concomitant chemotherapy group (Figs. 1 and 2), maintaining a comparable low toxicity level (Table 4), as dosimetric characteristics (Table 3).

The recent results of the PACIFIC trial point to a consistent benefit in the progression free survival when treatment using immunotherapy (durvalumab) is started within 1 to 42 days after the end of the concomitant chemoradiotherapy [29]. With the changing landscape in the standard treatment of LA-NSCLC, the reduction in treatment-induced toxicity, while maintaining optimal tumour control, has become a priority, thereby warranting access to adjuvant immunotherapy for these patients. The benefits of concomitant chemotherapy could be re-evaluated in this context. The use of immunotherapy concomitant to radiotherapy may be the next step [30]. Nevertheless, as immune cells are highly sensitive to conventional RT doses, the paradigm of the standard irradiation volumes should be reconsidered [31, 32]. In this context, the introduction of IMRT to spare lymphatic tissues and bone marrow deserves evaluation in prospective trials [33].

Our study has some limitations. Due to its retrospective nature, the prevalence of toxicity may be underestimated. The population was heterogeneous but constituted mainly by locally advanced stages and some selected stages IV (11%) and II (11%). Stage IV include only patients with one lesion in one anatomical site treated with radical intent as these patients are recognized with a better prognosis than multimetastatic patients [34]. Stages II include patients with bulky centrally located disease, non-operable and non-eligible to stereotactic radiotherapy.

Moreover, although our results pertaining to clinical outcomes are promising, this data only serve as a hypothesis generating resource.

## Conclusion

The use of accelerated IMRT opens a therapeutic window for radiation dose optimization for lung cancer, as an alternative to classical dose escalation strategies. The encouraging toxicity profile and efficacy of this technique warrants further investigation in a prospective clinical trial, especially in combination with immunotherapy.

## Abbreviations

BED: Biological effective dose
CI: Confidence interval
CT: Computed tomography
CTV: Clinical target volume
GTV: Gross tumor volume
H-RT: Hypofractionated radiotherapy
IMRT: Intensity modulated radiotherapy
ITV: Internal target volume
LA-NSCLC: Locally advanced non-small cell lung cancer
LRC: Loco-regional control
OS: Overall survival
OTT: Overall treatment time
PTV: Planning target volume
RT: Radiotherapy
SMART: Simultaneous modulated accelerated radiotherapy
